# Melkersson-Rosenthal syndrome and upadacitinib: A case report

**DOI:** 10.1016/j.jdcr.2025.12.002

**Published:** 2025-12-11

**Authors:** Donovan B. Turpin, Lee H. Grafton

**Affiliations:** a School of Medicine, Louisiana State University Health Sciences Center, Shreveport; b Grafton Dermatology and Cosmetic Surgery, Houma, Louisiana

**Keywords:** facial palsy, facial paralysis, fissured tongue, granulomatous cheilitis, lingua plicata, Melkersson-Rosenthal, Miescher cheilitis, orofacial edema, Rinvoq, upadacitinib

## Introduction

Melkersson-Rosenthal syndrome (MRS) is a rare disease with an unknown cause, classically presenting with a triad of peripheral facial palsy, lingua plicata (“fissured tongue”), and orofacial edema. Symptoms of MRS have an episodic relapsing-remitting course, which can progressively worsen. Facial swelling in MRS commonly involves the lips, and biopsy, showing noncaseating granulomas can help to confirm a diagnosis of MRS and rule out differential diagnoses, such as recurrent angioedema or infectious agents.^1^

Literature on pharmacotherapy for MRS is highly limited by low disease prevalence. The paucity of documented treatment options is typically immunosuppressive in nature. The most commonly reported medications used are glucocorticoids, which have shown mixed results in efficacy where literature is available.^1^^,^^2^ Case reports using other immunosuppressants or antimicrobial agents have been published as well.^2^^,^^3^ There is limited evidence on the usage of biologic agents, although there are reports of a few cases of MRS treated successfully with inhibitors of tumor necrosis factor alpha, a major cytokine involved in granuloma formation.^4^^,^^5^ Crohn’s disease, known for granulomatous pathology, is commonly treated with tumor necrosis factor alpha inhibitors.

Upadacitinib is a small molecule Janus kinase (JAK) inhibitor. Other JAK inhibitors are already widely used for granulomatous disorders ranging from cutaneous sarcoidosis to granuloma annulare.^6^ A case series (*n* = 5) has described a positive response to upadacitinib in granulomatous cheilitis (GC), a disease consisting of the same lip swelling as MRS without signs of lingua plicata or facial paralysis.^7^ Originally approved in 2019 by the Food and Drug Administration for refractory rheumatoid arthritis, upadacitinib has since been approved for treatment of several other inflammatory conditions. We present the case of a patient with the classic MRS triad, treated with upadacitinib.

## Case presentation

**Fig 1:**
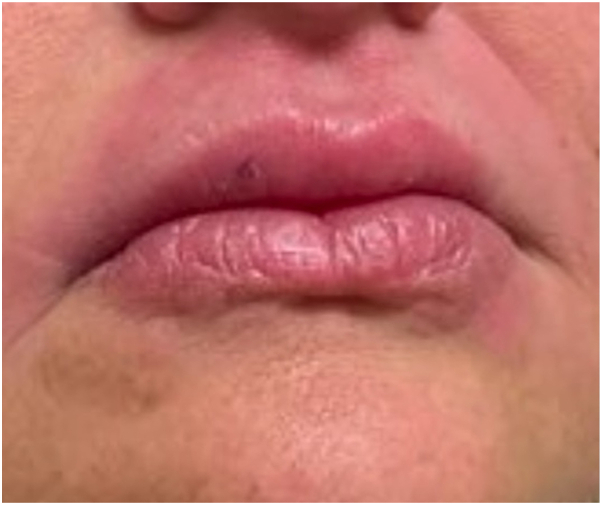
Swollen lips with perioral erythema; *red* edematous lesion on right upper lip.

**Fig 2:**
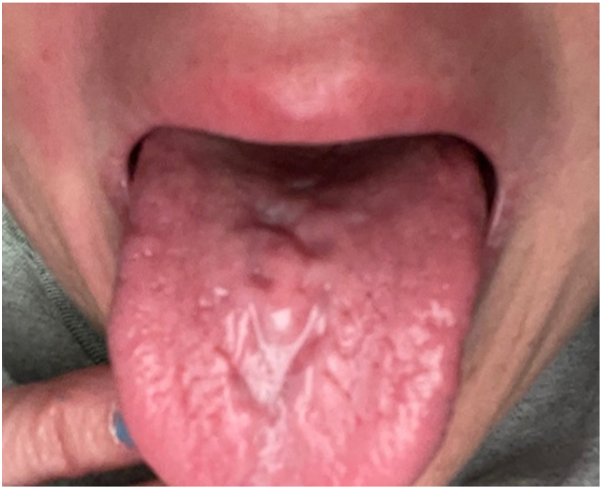
Lingua plicata, with pitting of median sulcus.

The patient, a 46-year-old White female, was referred to a dermatology clinic for recurrent episodes of facial swelling that had been refractory to other treatments, including glucocorticoid therapy. She reported a sensation of unilateral facial droop preceding most episodes, ipsilateral to the onset of swelling. Other history was significant for steroid-resistant atopic dermatitis and rosacea currently treated with doxycycline. Physical examination showed painful, diffusely enlarged lips with perioral erythema, along with red cheeks from rosacea. An edematous red spot with subtle nodularity was noted within the vermilion border of the upper lip (see Fig 1), and a punch biopsy was performed, which showed noncaseating granulomatosis. Additionally, the patient’s tongue was highly fissured, with deep pitting of the median sulcus (see Fig 2). The biopsy results, along with presence of the classic triad of symptoms, confirmed a diagnosis of MRS.

The patient showed no improvement of either orofacial swelling or atopic dermatitis after a month of treatment with dupilumab (a biologic agent blocking the common α-subunit of receptors for interleukin-4 and interleukin-13). She was switched to 15 mg oral upadacitinib once daily after counseling and blood tests.

**Fig 3:**
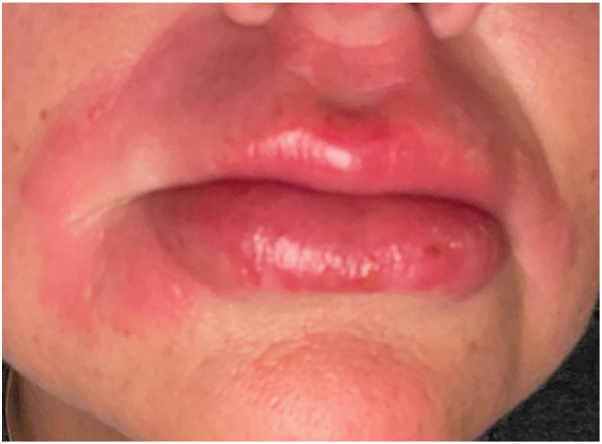
Breakthrough MRS flare from medication interruption. *MRS*, Melkersson-Rosenthal syndrome.

**Fig 4:**
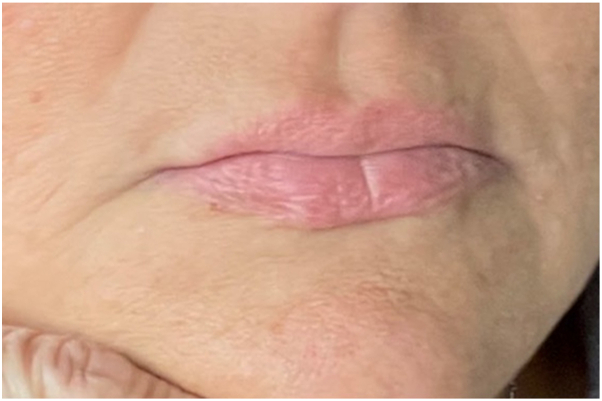
Resolution of swelling and erythema during upadacitinib treatment.

One month after starting treatment with upadacitinib, the patient returned to the clinic with improvement of lip swelling. During the first month, the patient experienced a single episode of herpes simplex virus (HSV) reactivation, presenting as a cold sore. She was prescribed a 1-month course of valacyclovir for treatment and continued using upadacitinib. Over the following year, she reported no further HSV reactivation, facial paralysis, or orofacial swelling while maintained on upadacitinib. During a period without access to a prescription refill, a breakthrough MRS flare occurred (see Fig 3), which resolved on resuming the upadacitinib regimen. Aside from the initial HSV breakout, the patient reported no adverse effects. Clinical examination after 4 months of treatment showed complete remission from swelling and erythema (see Fig 4).

## Discussion

This case illustrates the potential utility of upadacitinib for MRS. Literature on therapeutic options for MRS remains limited; therefore, it is crucial to continue exploring new treatments, especially for steroid-resistant cases like this. The patient’s disease progression showed a dramatic positive response to upadacitinib, which the patient tolerated well. It should be noted that doxycycline, which this patient was taking for another condition, has been used as adjunctive therapy to glucocorticoids in one MRS case.^3^

The usage of JAK inhibitors in MRS is supported by their usage across a broad range of granulomatous pathologies, with extensive evidence from the literature (see Table I). This research, along with several ongoing clinical trials, demonstrates vigorous active exploration in this field. Moreover, the case series on usage of upadacitinib in GC suggests potential utility due to the neighboring pathogeneses of GC and MRS. Our patient’s response, along with the current standing of the literature, demonstrates a need for further investigation on treating MRS with small molecule inhibitors and biologic agents.

**Table I:** JAK inhibitor usage in granulomatous diseases

Disease	JAK inhibitor	Articles	Study size (*n*)
Cutaneous sarcoidosis	Tofacitinib	Damsky et al, 2018	1
		Damsky et al, 2020	3
		Damsky et al, 2022∗	10
	Ruxolitinib	Rotenberg et al, 2018	1
		Levraut et al, 2019	1
		Wei et al, 2019	1
Multiorgan sarcoidosis	Tofacitinib	Damsky et al, 2019	1
		Friedman et al, 2021∗	5
Granuloma annulare	Tofacitinib	Damsky et al, 2019 Damsky et al, 2019	1
		Damsky et al, 2019	1
Granulomatous cheilitis	Upadacitinib	De Greef et al, 2024	5
Necrobiosis lipoidica	Ruxolitinib	Lee et al, 2018	1
	Tofacitinib	Damsky et al, 2019	1
Hemophagocytic lymphohistiocytosis	Ruxolitinib	Broglie et al, 2017	1
		Slostad et al, 2018	1
		Zandvakili et al, 2018	1
		Ahmed et al, 2019∗	5
		Sin et al, 2019	1
		Wang et al, 2019	1
		Zhao et al, 2019	1
Crohn’s disease	Upadacitinib	Loftus et al, 2023∗	1523
		Wu et al, 2025∗	21
Giant cell arteritis	Upadacitinib	Blockmans et al, 2025∗	209

*JAK*, Janus kinase.

∗ Registered clinical trial.

## Conflicts of interest

None disclosed.

## References

[1] Elias M.K., Mateen F.J., Weiler C.R. (2013). The Melkersson–Rosenthal syndrome: a retrospective study of biopsied cases. J Neurol.

[2] Wehl G., Rauchenzauner M. (2018). A systematic review of the literature of the three related disease entities cheilitis granulomatosa, orofacial granulomatosis and Melkersson-Rosenthal syndrome. Curr Pediatr Rev.

[3] Oudrhiri L., Chiheb S., Marnissi F., Zamiati S., Benchikhi H. (2012). Successful treatment of Miescher's cheilitis in Melkersson-Rosenthal syndrome with betamethasone injections and doxycycline. Pan Afr Med J.

[4] de Moll E.H., Lebwohl M.G. (2018). Melkersson-Rosenthal syndrome successfully treated with adalimumab. Cutis.

[5] Stein J., Paulke A., Schacher B., Noehte M. (2014). An extraordinary form of the Melkersson-Rosenthal syndrome successfully treated with the tumour necrosis factor-α blocker adalimumab. BMJ Case Rep.

[6] Wang A., Singh K., Ibrahim W., King B., Damsky W. (2020). The promise of JAK inhibitors for treatment of sarcoidosis and other inflammatory disorders with macrophage activation: a review of the literature. Yale J Biol Med.

[7] De Greef A., Peeters C., Dewit O., de Montjoye L., Baeck M. (2024). Upadacitinib for treatment of granulomatous cheilitis. JAMA Dermatol.

